# National 10-m soybean maps for South Africa from 2018 to 2025

**DOI:** 10.1038/s41597-026-07295-z

**Published:** 2026-04-28

**Authors:** Xin Huang, Anton Vrieling, Yue Dou, Francis Muthoni, Adolph Nyamugama, Adeniyi Adeyemi, Terry Newby, Andrew Nelson

**Affiliations:** 1 Faculty of Geo-Information Science and Earth Observation (ITC), University of Twente, 7500 AE Enschede, the Netherlands; 2 Farming System Agronomy Program, International Institute of Tropical Agriculture, Box, 30772-00100 Nairobi, Kenya; 3 Agricultural Research Council-Natural Resource & Engineering (ARC-NRE), 600 Belvedere street-Arcadia, Pretoria, Republic of South Africa; 4 GeoTerraImage (Pty) Ltd., Building C (Ground Floor), Agri Hub Business Park, 477 Witherite Street, 0184 Pretoria, South Africa

## Abstract

Accurate and spatially detailed information on soybean cultivation is essential for production estimation and land-use management, particularly as cultivated areas expand in emerging producing regions such as South Africa. This research presents SoySA10, a national 10-m soybean mapping dataset for South Africa covering 2018–2025. The dataset was generated from Sentinel-2 time series using a generalised classification model – combining random forest with dynamic time warping – with spectral-phenological features. SoySA10 includes time-series soybean distribution maps, field type maps, and a soy-cultivation change map. Accuracy assessment showed an overall accuracy of 0.89, with F1-scores of 0.66 for soybean and 0.94 for non-soybean classes. Comparisons between mapped and reported soybean areas exhibited high consistency at provincial (R^2^ = 0.97 and nRMSE = 7%) and district levels (R^2^ > 0.80 and nRMSE < 20%). Strong spatial agreement was observed between SoySA10, the Spatial Production Allocation Model (SPAM) 2020, and soybean field boundary polygons at both aggregate and detailed scales. The dataset supports analyses of South Africa’s soy-cultivation dynamics and related downstream applications.

## Background & Summary

As a major source of energy, protein, and oil for both animal feed and human food, soybean (*Glycine max*) plays an important role in meeting the rising global food demand^1^. Approximately 76% of global soybean production is processed for animal feed^2^, and meat consumption is projected to continue increasing due to population growth and rising demand^3^. While growing demand and higher prices for farmers stimulate the expansion of soybean cultivation, this trend also poses threats to ecosystems (e.g., deforestation in the Amazon)^4–7^ and may lead to the displacement of staple food crops^8^. Accurate monitoring of the spatial and temporal dynamics of soybean cultivation is therefore essential for balancing agricultural production with the preservation of ecosystem services^9^.

Remote sensing (RS) is a powerful tool for soybean mapping, particularly with the advances in freely available satellite datasets at 10–30 m spatial resolution (e.g., Sentinel-2 and Landsat series) and the development of planetary-scale cloud computing platforms (e.g., Google Earth Engine). Multiple crop mapping products and datasets are now available for major soybean-producing countries or regions. For example, the United States crop data layer (CDL) provides annually updated 30-m crop type maps^10^. Song *et al*.^7^ monitored soybean expansion in South America over the past two decades using Landsat imagery. Based on Sentinel-2 time series, three national-scale soybean mapping efforts have been conducted in China. Li *et al*.^11^ generated a maize–soybean map for 2019, while Mei *et al*.^12^ and Zhang *et al*.^13^ produced time-series soybean maps covering 2017–2021 and 2019–2022, respectively. These datasets support the monitoring of soybean cultivation in major producing regions. However, current datasets are far from geographically comprehensive, with few soybean mapping efforts existing for African countries. Soybean is an important commodity crop for local consumption and trade in Africa, where the harvested area has increased fivefold over the past 20 years^14^. Moreover, due to its high protein content and rising market prices, several African countries have advocated increasing local soybean production in recent years^15^.

Producing accurate RS-based crop maps for many African countries is challenging. For general cropland mapping, Kerner *et al*.^16^ compared 11 existing land cover datasets and found very low agreement in cropland distribution among them. The average F1-score for cropland was below 0.70 in seven of eight target countries. A more recent study by Lou *et al*.^17^ produced a valuable cropland extent dataset for Africa using 30-m Landsat imagery, with F1-scores around 0.60 across the assessed countries. For soy-specific maps, to our knowledge, no public RS-based soybean maps exist for African countries. Huang *et al*.^18^ conducted a soybean mapping study across ten global sites, including in Burkina Faso and Zambia. However, the F1-scores for these two sites (<0.60) were substantially lower than those in other countries (>0.80). The limited classification accuracy of cropland and soybean in Africa can be attributed to various challenges, including heterogeneous landscapes, dynamic agricultural calendars, the prevalence of weeds and pests, sub-optimal field management, intercropping, and the high diversity of cultivated crops^19–21^. To better understand the impacts of field characteristics on crop mapping accuracy, Huang *et al*.^22^ categorised crop fields by field area and water stress and found divergent mapping accuracies across different field types. Their results highlight the importance of providing field type information for more detailed and reliable analysis of crop type maps in smallholder farming regions.

South Africa is a major soy-cultivating country in Africa, contributing 23% of the continent’s soybean cultivation area^14^. Moreover, the country has experienced a substantial increase (46%) in soybean cultivating area from 2018 to 2024^23^ and is transitioning from a soybean net importer to a net exporter^24^. Nonetheless, to our knowledge, no public RS-based soybean maps exist for South Africa at a high spatial resolution. To fill this gap, we aimed to produce reliable soybean maps for South Africa at 10 m spatial resolution (SoySA10), spanning the 2017/18 to 2024/25 crop growing seasons. Such maps are not only helpful for tracking commercial soybean expansion but are also fundamental to improving the income and food security of smallholder farmers, both of which co-exist and are vital to South Africa’s agricultural sector.

## Methods

**Fig. 1:**
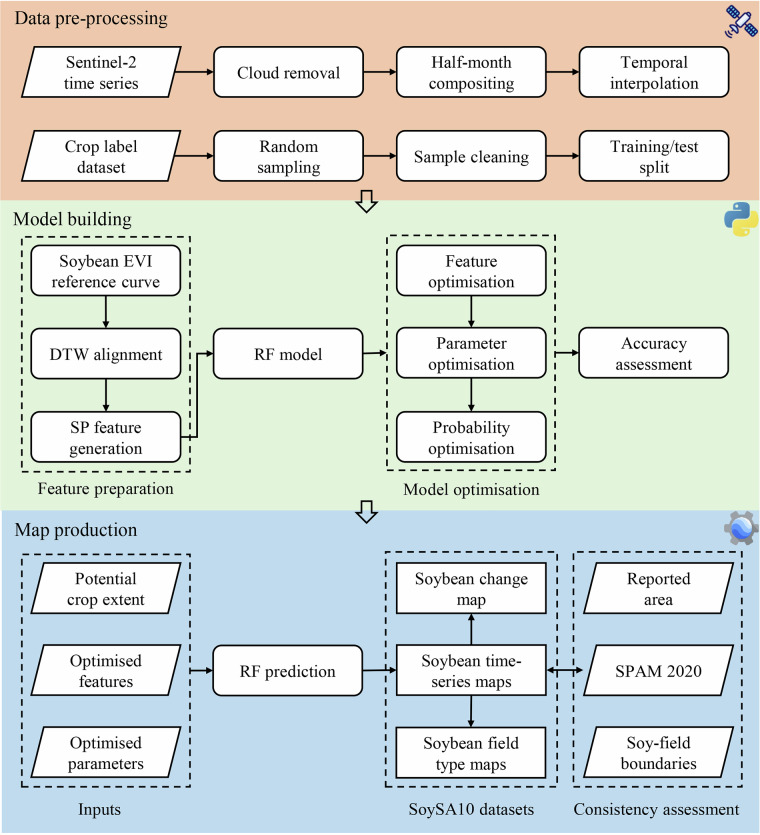
Workflow for producing SoySA10. EVI: enhanced vegetation index, DTW: dynamic time warping, SP: spectral-phenological, RF: random forest, and SPAM: spatial production allocation model.

We first applied a robust soybean mapping method, combining random forest with dynamic time warping (RF-DTW)^18^, to generate soybean distribution maps based on Sentinel-2 imagery. RF-DTW was proposed by Huang *et al*.^18^ to address spatio-temporal phenological variations across large areas where crop fields exhibit diverse growth progress due to varying climate conditions and agricultural practices. The resulting maps were then validated for accuracy and consistency using multi-source datasets. Based on the distribution maps and insights from crop field categorisation in Huang *et al*.^22^, we used a water stress index to categorise soybean fields into different types, generating field type maps that provide supplementary information on classification confidence and field management. Finally, we provided information on changes in soybean cultivation areas and field types in South Africa over recent years. The RF model was optimised locally (Python, scikit-learn 1.5.2) and implemented it on the GEE cloud computing platform. The workflow is summarised in Fig. 1. Details of data pre-processing, model building, and map production procedures are described in the following subsections.

### Study area

**Fig. 2:**
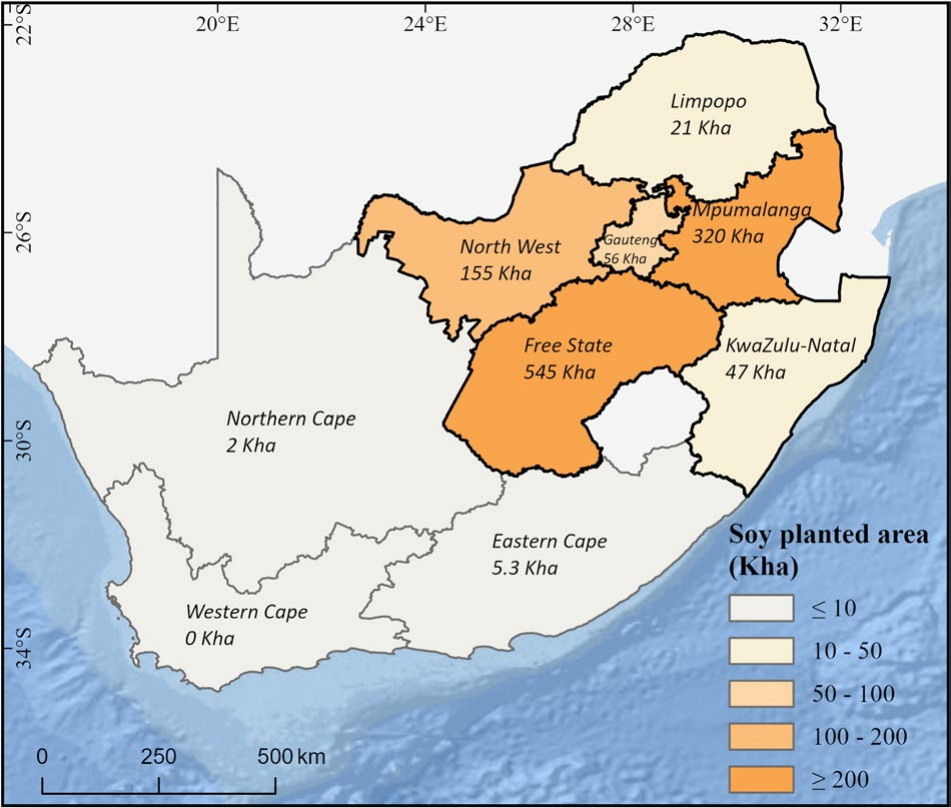
Soybean planted area in South Africa in 2024. Data source: Crop Estimate Committee^23^.

South Africa is the second-largest soy-cultivating country in Africa, with a cultivated area of 1,150 Kha in 2024 (throughout this study, year references correspond to the harvest year), representing a substantial increase from 787 Kha in 2018^23^. The soybean growing season extends from November to May and is mainly cultivated in the north-eastern part of the country (Fig. 2). The major producing provinces are: Free State (FS), Mpumalanga (MP), North West (NW), Gauteng (GP), KwaZulu-Natal (KZN), and Limpopo (LP). These six provinces accounted for 99.4% of the country’s total soybean area^23^ and were selected as the target region for soybean mapping in this study.

### Satellite data

We used Sentinel-2 Level-1C data (“COPERNICUS/S2_HARMONIZED”) for soybean mapping. We chose Level-1C instead of Level-2A because the Level-2A coverage is incomplete for our study area prior to 2019 on the Google Earth Engine (GEE) platform. Furthermore, Level-1C data are proven suitable and have been widely used for crop classification in previous large-scale studies^12,25,26^. Sentinel-2 provides multi-spectral imagery with 10/20/60 m spatial resolution and a global revisit frequency of 5 days from 2017 onwards. Nine relevant spectral bands were selected for classification: B2 (blue), B3 (green), B4 (red), B5 (red edge 1), B6 (red edge 2), B7 (red edge 3), B8 (near-infrared), B11 (shortwave infrared 1), and B12 (shortwave infrared 2). The bands with 20 m resolution (i.e., B5, B6, B7, B11, and B12) were resampled to 10 m resolution using the nearest neighbour resampling method. Time-series data covering the soybean growing season from 15 November of the planting year to 15 May of the harvest year were processed for soybean mapping. The mapping period spans from the 2017/18 to the 2024/25 growing seasons. Sentinel-2 data were pre-processed in three steps: (1) cloud removal using the Google Cloud Score dataset (“GOOGLE/CLOUD_SCORE_PLUS/V1/S2_HARMONIZED”) in GEE, with pixels having a clear score below 0.50 masked out; (2) half-month image compositing based on the maximum ratio of near-infrared to blue reflectance (RNB)^27^, to standardize temporal intervals and generate high-quality spectral reflectance data; and (3) temporal linear interpolation to fill gaps in the time-series caused by persistent cloud cover^18,28^.

### Reference crop label dataset

A crop field boundary (CFB) dataset (.shp), containing accurately delineated fields and crop type labels for Free State from the 2018 to 2021 harvest years, was obtained through a data request to South Africa’s Department of Agriculture, Land Reform and Rural Development (DALRRD). The dataset was generated by local experts using aerial imagery, Sentinel-2 time series, and a crop growth calendar. Approximately 4,500 field polygons representing crops, pasture, and fallow land were randomly sampled from this dataset. A 20-m inner buffer was applied to each field polygon to reduce mixed pixels and edge effects, and one point was then randomly generated within each field to avoid same-field leakage and create a reference crop label dataset for model training and accuracy assessment. The major crops included maize, soybean, groundnuts, sunflower, and wheat. To ensure data quality, three criteria based on the enhanced vegetation index (EVI) time series were applied to retain reliable crop samples: (1) 15_th_ percentile of EVI ≥ 0 and maximum EVI ≥ 0.3 (indicating active cultivation), (2) EVI amplitude ≥ 0.2 (indicating crop growth), and (3) minimum EVI ≤ 0.4 (indicating crop maturity).

For other natural vegetation (e.g., forest, shrub) and non-vegetation types (e.g., bare soil, water, and built-up), we incorporated 570 samples from the global land cover estimation (GLanCE) project^29^ and generated an additional 581 samples through visual interpretation of Google Earth imagery and Planet monthly basemaps during the corresponding years. In total, 5,457 samples were compiled (Table 1) and randomly split into training and test datasets (1:1) for classification model building and accuracy assessment, respectively.

**Table 1:** Constructed reference crop label dataset.

Type	2018	2019^*^	2020	2021	All
Soybean	190	358	189	189	926
Maize	284	561	289	287	1,421
Sunflower	147	266	146	130	689
Groundnuts	85	179	99	97	460
Wheat	50	78	55	73	256
Other crops	28	46	30	29	133
Natural vegetation	369	365	302	92	1,128
Non-vegetation	285	80	40	39	444
Total	1,438	1,933	1,150	936	5,457

^*^2019 was a drought year; therefore, the number of crop samples collected from that year was twice that of other years to improve model generalisation.

### Reported area

Reported soybean planted areas at the provincial level (2018–2025) and the magisterial district level (2018–2024) were obtained from South Africa’s Crop Estimate Committee (CEC). Provincial-level areas were estimated by CEC using a statistical survey, in which questionnaires were sent to a sample of producers at the start of the season, and through helicopter-based aerial surveys, with survey points statistically selected. Official provincial-level data were published for 2018–2025 (https://www.sagis.org.za/crop-estimates-committee-2/). District-level areas for FS and NW were sourced from GeoTerraImage^30^, which were estimated based on their commercial crop type classification maps, including soybean derived from Sentinel-1 and -2 imagery, with a reported F1-score for soybean higher than 0.80.

### SPAM 2020

As no public RS-based soybean maps currently exist for South Africa, the soybean harvest area map from the spatial production allocation model (SPAM) 2020^31^ was selected to compare the overall soybean distribution at the national level with SoySA10. SPAM provides global spatial patterns of crop production and harvested areas at a 10 × 10 km resolution. It employs a cross-entropy method to generate plausible estimates of crop distribution based on various inputs, such as (sub)national statistics, land cover maps, and crop-specific suitability information^32^. SPAM maps effectively capture crop spatial distribution patterns at large scales and have been widely used in agriculture-related research and applications^33,34^.

### Classification feature generation

**Fig. 3:**
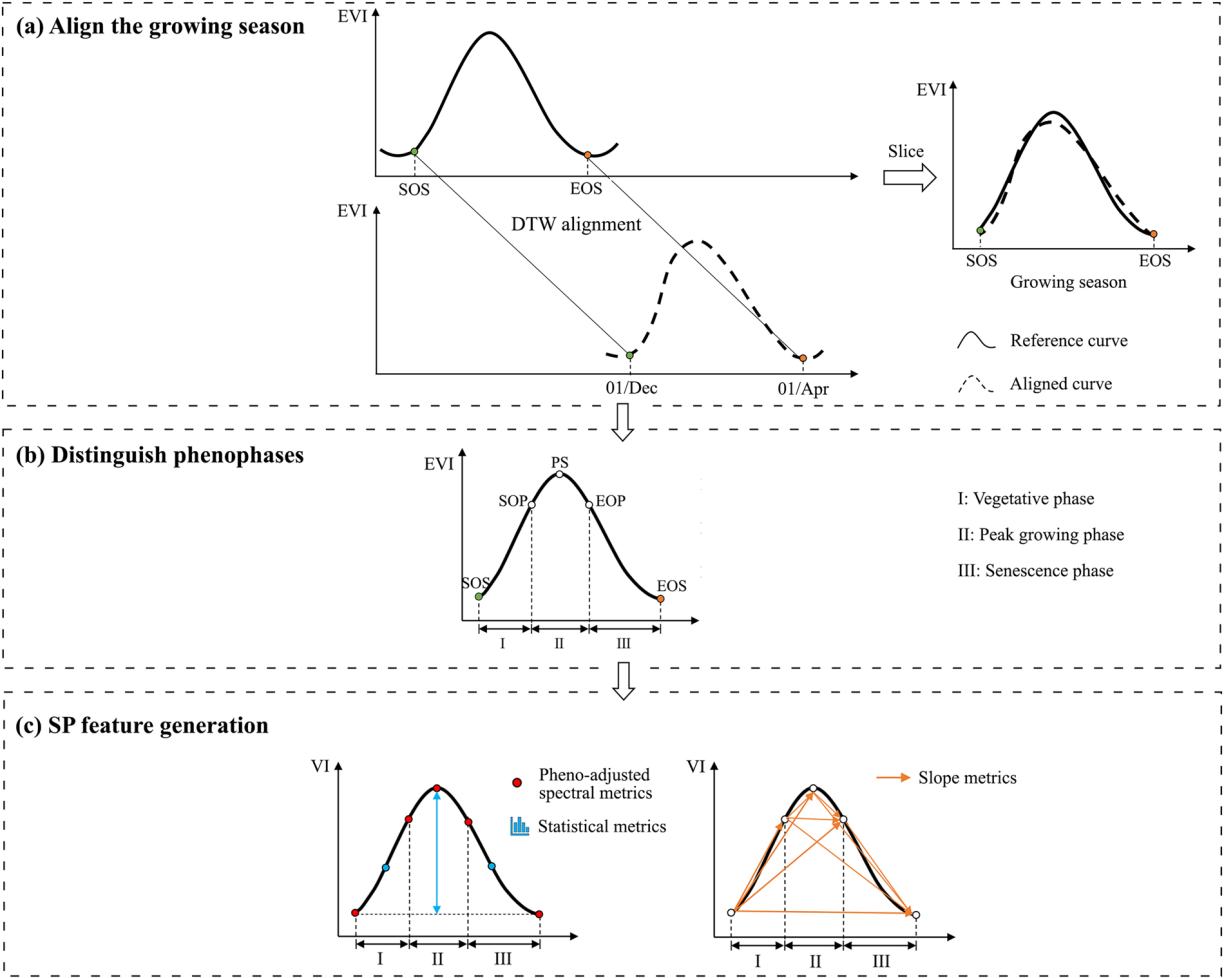
Workflow for generating spectral-phenological (SP) features. DTW: dynamic time warping, SOS: start of season, SOP: start of peak season, PS: peak of season, EOP: end of peak season, and EOS: end of season. Figure adapted from Huang *et al*.^18^.

RF-DTW first uses dynamic time warping (DTW) to phenologically align time series and then generates robust spectral-phenological (SP) features to be used in RF models (Fig. 3). Specifically, the EVI time-series curves from soybean training samples were averaged to generate a reference soybean curve, with the start of season (SOS) and end of season (EOS) points defined based on EVI values and curve shapes (Fig. 3a). Then, the EVI time-series curve for each pixel (whether soybean or another crop or land cover) was aligned to the reference curve, and the corresponding SOS and EOS points of the aligned curve were identified. The time series between SOS and EOS (i.e., the growing season) was retained for further processing. Within the growing season, the point with maximum EVI value was defined as the peak of season (PS) point, and three phenophases were distinguished (Fig. 3b):

- Peak growing phase: three half-month composites comprising the PS point and the composites immediately before (start of peak season, SOP) and after (end of peak season, EOP).
- Vegetative phase: from SOS to SOP;
- Senescence phase: from EOP to EOS.
  Based on the distinguished phenophases, three categories of SP features were derived (Fig. 3c):
- Pheno-adjusted spectral metrics (5 metrics): spectral reflectance and vegetation indices (VIs) for each phenological point (i.e., SOS, SOP, PS, EOP, EOS);
- Statistical metrics (6 metrics): (i) mean of spectral reflectance or VIs during each phenophase; (ii) mean, standard deviation, and amplitude of spectral reflectance or VIs during the whole growing season;
- Slope metrics (10 metrics): slopes of spectral reflectance and VIs between each phenological point.

A total of 357 SP features were generated from 9 spectral bands and 8 VIs (Table 2), i.e., (5 pheno-adjusted metrics + 6 statistical metrics + 10 slope metrics) × (9 spectral bands + 8 VIs). For further details of the RF-DTW method and SP feature generation, we refer to Huang *et al*.^18^.

**Table 2:** Vegetation indices used in this study (ordered alphabetically).

Vegetation Index	Formula	Reference
Green Chlorophyll Vegetation Index (GCVI)	$\frac{{B}_{8}}{{B}_{3}}-1$	Gitelson *et al*.^53^
Greenness and Water Content Composite Index (GWCCI)	$\frac{{B}_{8}-{B}_{4}}{{B}_{8}+{B}_{4}}\times {B}_{11}$	Chen *et al*.^54^
Greenness and Water Content Composite Index 2 (GWCCI2)	${EVI}\times {NDSVI}$	Huang *et al*.^55^
Enhanced Vegetation Index (EVI)	$2.5\times \frac{{B}_{8}-{B}_{4}}{{B}_{8}+{6B}_{4}-7.5{B}_{2}+1}$	Huete *et al*.^56^
Normalized Difference Senescent Vegetation Index (NDSVI)	$\frac{{B}_{11}-{B}_{4}}{{B}_{11}+{B}_{4}}$	Qi *et al*.^57^
Normalized Difference Tillage Index (NDTI)	$\frac{{B}_{11}-{B}_{12}}{{B}_{11}+{B}_{12}}$	Van Deventer *et al*.^58^
Red Edge Position (REP)	$705+35\times \frac{0.5\times {(B}_{7}+{B}_{4})-{B}_{5}}{{(B}_{6}-{B}_{5})}$	Defourny *et al*.^59^
Short-wave Infrared Water Stress Index (SIWSI)	$\frac{{B}_{11}-{B}_{8}}{{B}_{11}+{B}_{8}}$	Fensholt and Sandholt^60^

### Classification feature optimization

**Fig. 4:**
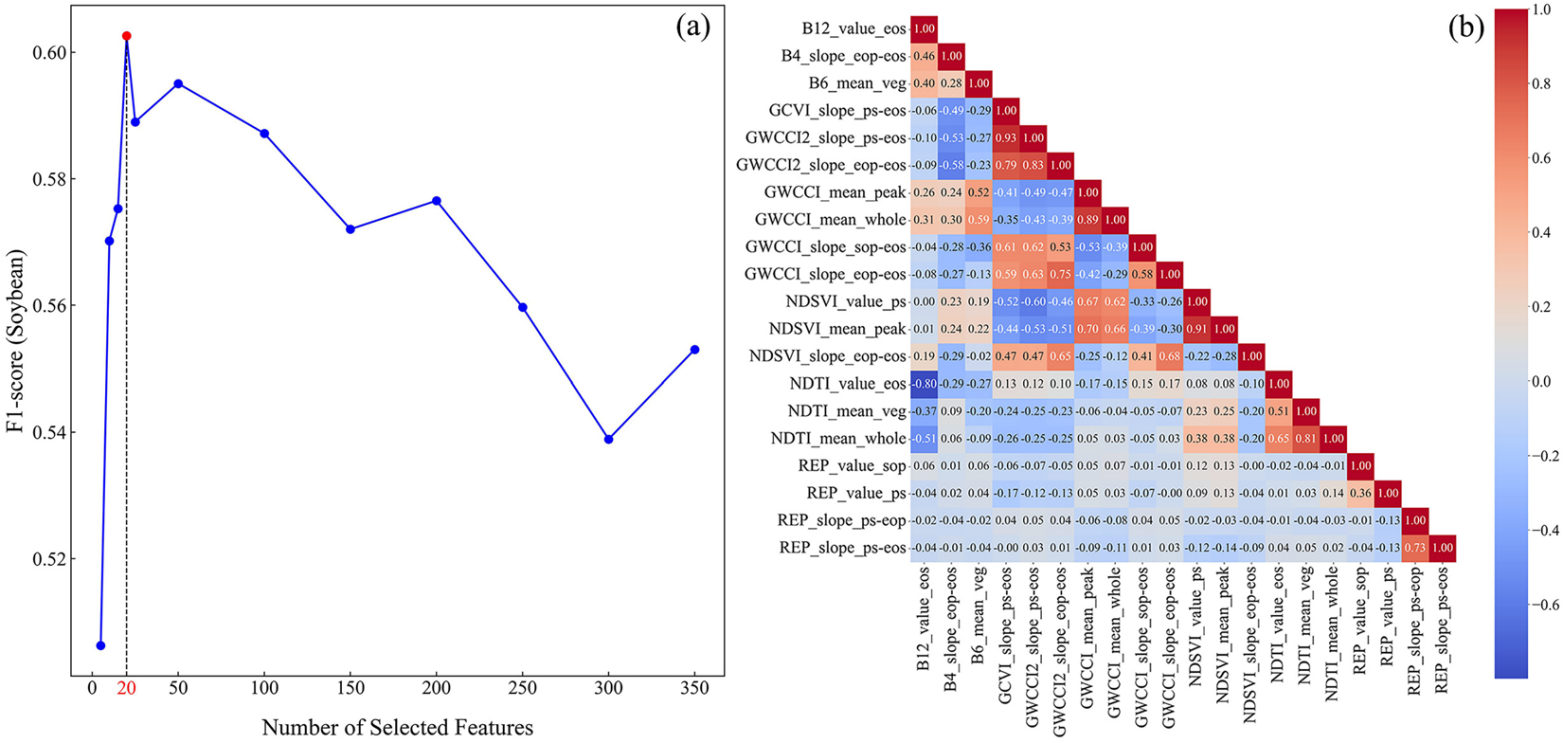
Cross-validated F1-scores for soybean classification using the recursive feature elimination algorithm with different numbers of selected features (**a**), and the correlation coefficient matrix (**b**) for the 20 most important features. The format of the feature names is explained in the caption of Table 3.

Using a large number of features can cause redundancy and be inefficient for mapping. To optimise the feature set, we used the recursive feature elimination (RFE)^35^ with five-fold cross-validation based on the RF classifier and training samples. The 20 most important features achieved the highest F1-score for soybean (Fig. 4a). A correlation matrix was generated for these 20 features (Fig. 4b). To reduce feature correlation, the latter feature in each highly correlated pair (|r| > 0.8, p < 0.05) was removed, resulting in the final set of 16 features (Table 3).

**Table 3:** Optimised classification feature set.

Category	Feature
Pheno-adjusted spectral metrics	*B12_value_eos, NDSVI_value_ps, NDTI_value_eos, REP_value_sop, REP_value_ps*
Statistical metrics	*B6_mean_veg, GWCCI_mean_peak, NDTI_mean_veg*
Slope metrics	*B4_slope_eop-eos, GCVI_slope_ps-eos, GWCCI2_slope_eop-eos, GWCCI_slope_sop-eos, GWCCI_slope_eop-eos, NDSVI_slope_eop-eos, REP_slope_ps-eop, REP_slope_ps-eos*

*The feature name format is *Band/VI_metric_period*. For example, *B12_value_eos* indicates the B12 value at the end-of-season (eos) point; *B6_mean_veg* indicates the mean value of B6 during the vegetative phase; and *B4_slope_eop-eos* indicates the slope of B4 between the end-of-peak (eop) point and the eos point.

### RF parameter optimization

**Fig. 5:**
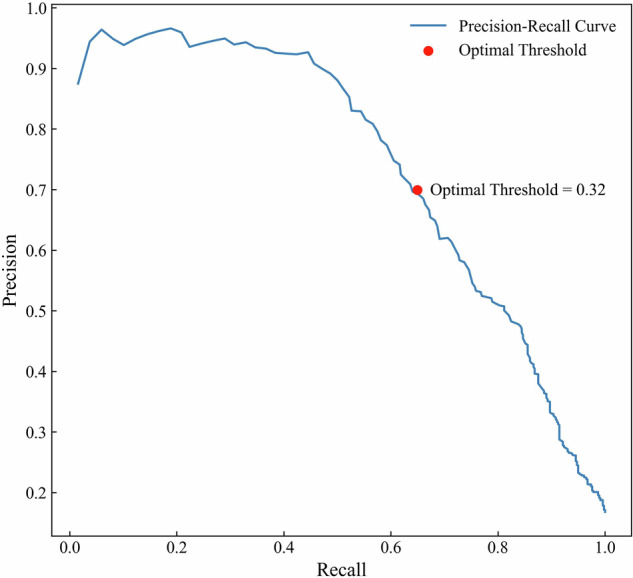
Precision-Recall curve for soybean classification across varying probability thresholds.

We built a binary RF model (soybean *vs*. non-soybean) based on the optimised feature set (Table 3), and used five-fold cross-validation on the training samples for RF parameter optimisation. The major parameters were tuned using the successive halving algorithm^36^, which iteratively halves the number of parameter configurations while allocating more computational resources to the better-performing ones. The parameter set was optimised via successive halving within constrained search spaces, as detailed in Table 4. The optimal threshold for soybean classification (i.e., pixels with RF probability above the threshold were classified as soybean) was identified using the precision-recall curve^37,38^ as the value yielding the highest F1-score, and was set to 0.32 (Fig. 5).

**Table 4:** Hyperparameter search space and optimised parameter set.

Parameter	Search Space	Optimised Value
numberOfTrees	[30, 50, 100, 150, 200, 300]	300
variablesPerSplit	[‘sqrt’, ‘log2’, 5, 6, 7, 8, 9, 10]	9
maxNodes	[30, 40, 50, 60, 70, 80, 90, 100, None]	100
bagFraction	[0.4, 0.5, 0.6]	0.6
minLeafPopulation	[1, 2, 4, 6, 8, 10]	1

### Map production

We used GEE for map production, including four major procedures:

1. Hexagon tile division: We divided the six soy-producing provinces into 156 hexagon tiles, each with a side-to-side diameter of 0.4°. To improve computational efficiency, we excluded 21 tiles with less than 1% cropland (based on ESA WorldCover 2021^39^), since these areas predominantly represent non-agricultural landscapes (e.g., arid land, forest, savanna, or natural vegetation) and are unlikely to support crop cultivation^40^.
2. Potential crop extent generation: The same criteria used for crop sample cleaning were applied to generate the potential crop extent. An additional criterion was added based on the EVI slice between aligned SOS and EOS points: the growing season length had to exceed 60 days (i.e., more than four composites), reflecting the minimum period required for soybean growth.
3. Soybean prediction: Based on the optimised features and parameters, the optimised RF model was implemented in GEE to predict soybean distribution within the potential crop extent. A majority filter with a 5 × 5 window was applied as a post-classification procedure to reduce salt-and-pepper noise in the resulting soybean maps^41,42^.
4. Soybean cultivation change detection: We generated a change map to provide spatially detailed information on soybean cultivation dynamics between the periods of 2018–2020 and 2023–2025. Given the common practice of crop rotation in soybean planting, a field was considered as a soybean field if soybean was cultivated at least once within either of the three consecutive-year periods. Soybean fields were then classified as: (i) new soybean fields, cultivated in 2023–2025 but not in 2018–2020; (ii) unchanged soybean fields, cultivated in both periods; and (iii) former soybean fields, cultivated with soybean in 2018–2020 but not in 2023–2025.

### Field type categorization

**Fig. 6:**
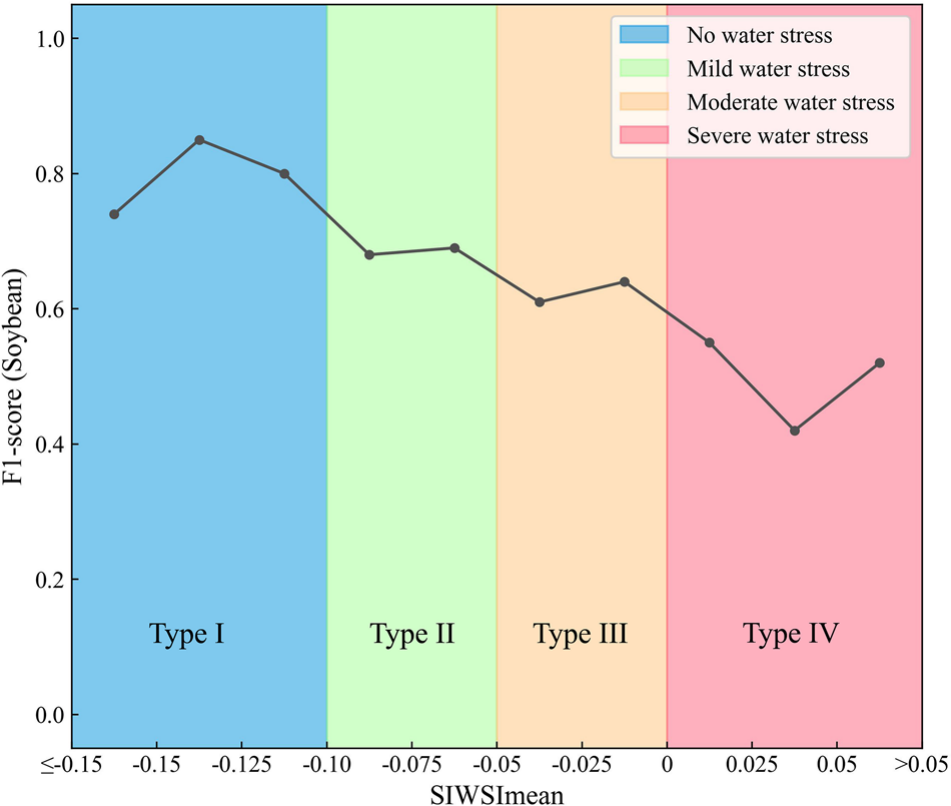
Soybean F1-scores assessed using test crop samples within different SIWSImean ranges.

Considering the varying crop management levels and the prevalent drought threat to crop growth in South Africa, we used the mean value of SIWSI (SIWSI_mean_) during the growing season to characterise crop water stress and categorise crops into different types. A higher SIWSI value indicates a higher level of water stress. Soybean F1-scores were assessed using test crop samples within different SIWSI_mean_ ranges, which showed sensitivity to mapping accuracy (Fig. 6). Based on the varying accuracy levels across SIWSI_mean_ values, we defined four field types: Type I – no water stress (SIWSI_mean_ ≤ −0.10), Type II – mild water stress (−0.10 < SIWSI_mean_ ≤ −0.05), Type III – moderate water stress (−0.05 < SIWSI_mean_ ≤ 0), and Type IV – severe water stress (SIWSI_mean_ > 0).

### Model classification accuracy assessment

We selected four commonly used classification accuracy measures, including precision, recall, F1-score and overall accuracy (OA) (Eqs. 1–4) to assess the classification model accuracy based on the test samples. We applied bootstrapping with replacement, repeating 5,000 times, to estimate the 95% confidence intervals for these measures^42,43^. In addition to the train-test-split accuracy assessment, we adopted leave-one-year-out (LOYO) and leave-one-region-out (LORO) cross-validation to assess the generalisation of the classification model. Specifically, LOYO iteratively used one year as the unseen test year and the remaining years for training until each year had been used for testing, after which the accuracies were averaged. Similarly, LORO was implemented by grouping 18 major soybean-cultivating magisterial districts in the Free State into eight regions, each iteratively held out for testing. Note that the model accuracies are not fully representative of the map accuracies across South Africa, as crop samples were collected only from the Free State. The consistency assessment at the national scale is further described in the following section of map consistency assessment.

1 $${\rm{Precision}}=\frac{{N}_{i}}{{C}_{i}}$$

2 $${\rm{Recall}}=\frac{{N}_{i}}{{T}_{i}}$$

3 $${{\rm{F}}}_{1}\,{\rm{score}}=2\times \frac{{\rm{Precision}}\times {\rm{Recall}}}{{\rm{Precision}}+{\rm{Recall}}}$$

4 $${\rm{Overall\; accuracy}}=\frac{{N}_{c}}{A}$$

where ${N}_{i}$ is the number of correctly classified test samples for class *i*; ${C}_{i}$ is the number of test samples classified as class *i*; ${T}_{i}$ is the number of test samples belonging to class *i*; ${N}_{c}$ is the total number of correctly classified test samples across all classes; *A* is the total number of all test samples.

### Map consistency assessment

We assessed map consistency using multi-source datasets: (1) comparing the mapped area, calculated with the*.pixelArea()* function in GEE to account for projection distortion, against the reported area at both provincial and district levels; (2) comparing the overall soybean distribution with the SPAM 2020 soybean harvest area map, for which our map was upscaled to the same 10 km spatial resolution for area statistics; and (3) comparing the detailed soybean distribution with the CFB dataset in Free State. We selected the following indicators to assess the consistency between mapped and reported areas: coefficient of determination (R^2^), root mean square error (RMSE), and normalised RMSE (nRMSE_s_ and nRMSE_t_) (Eqs. 5–8):

5 $${{\rm{R}}}^{2}=\frac{{{cov}(\hat{Y},Y)}^{2}}{{var}(\hat{Y}){var}(Y)}$$

6 $${\rm{RMSE}}=\sqrt{\frac{{\mathop{\sum }\limits_{i=1}^{N}({\hat{Y}}_{i}-{Y}_{i})}^{2}}{N}}$$

7 $${{\rm{nRMSE}}}_{{\rm{s}}}=\frac{{RMSE}}{{Y}_{\max }-{Y}_{\min }}\times 100{\boldsymbol{ \% }}$$

8 $${{\rm{nRMSE}}}_{{\rm{t}}}=\frac{{RMSE}}{\bar{Y}}\times 100{\boldsymbol{ \% }}$$

where ${\hat{Y}}_{i}$ is the mapped area of the *i*-th sample, *Y*_*i*_ is the reported area of the *i*-th sample, *Y*_*max*_, *Y*_*min*_, and $\bar{Y}$ are the maximum, minimum, and average reported areas, respectively, $\mathrm{cov}\,(\hat{Y},Y)$ is the covariance between the mapped and reported areas, and $\mathrm{var}\,\left(\hat{Y}\right)$ and *var* (*Y*) are the variances of the mapped and reported areas, respectively. nRMSE_s_ was used to normalise RMSE across space to avoid biasing the average toward (near) non-cultivating regions disproportionately. Conversely, nRMSE_t_ was used over time considering cases where the temporal trend remained stable.

## Data Records

The 10-m soybean mapping dataset for South Africa (SoySA10)^44^ is publicly available on Zenodo (10.5281/zenodo.17569053). The dataset includes annual soybean distribution maps (2018–2025), annual soybean field type maps (2018–2025), and a soybean cultivation change map comparing the periods 2018–2020 and 2023–2025, all at 10-m spatial resolution. In the distribution maps, pixel values of 0 and 1 represent non-soybean and soybean, respectively. In the field-type maps, values of 1, 2, 3, and 4 correspond to no water stress (type I), mild water stress (type II), moderate water stress (type III), and severe water stress (type IV), respectively. In the soybean cultivation change map, values of 0, 1, 10, and 11 represent non-soybean, former soybean, newly cultivated soybean, and unchanged soybean, respectively. All raster datasets are provided in GeoTIFF format using the WGS 1984 coordinate reference system (EPSG:4326). The reference crop label dataset for South Africa^44^ is also available on Zenodo (10.5281/zenodo.17569053) and is provided as a vector file containing crop type or land cover labels. All datasets can be visualised and analysed using geographic information system software such as ArcGIS or QGIS.

## Technical Validation

### Temporal spectral profiles

**Fig. 7:**
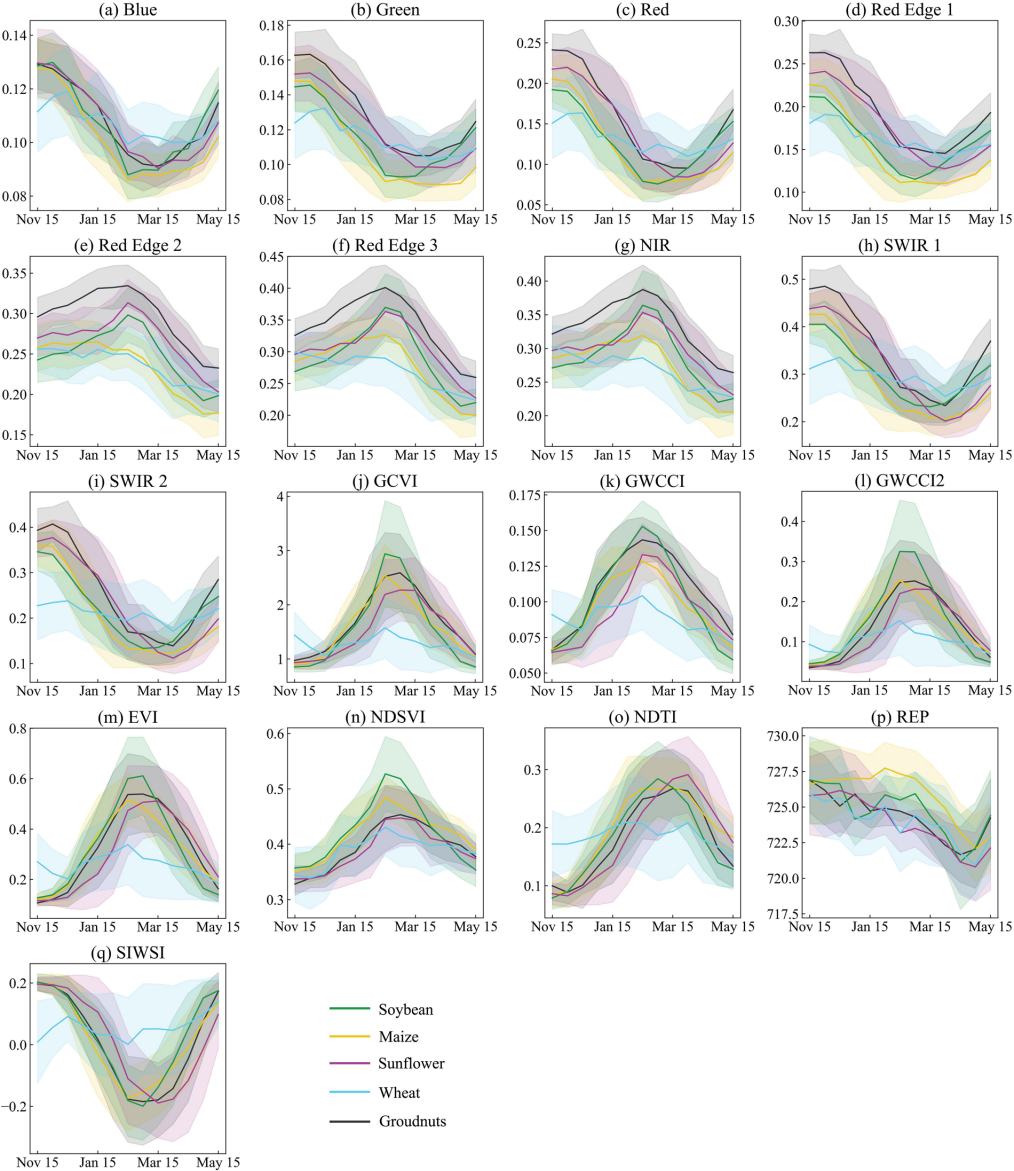
Temporal spectral profiles for soybean and other major crops, generated from training samples. Solid lines represent mean values calculated within the 10th–90th percentiles, and shaded areas indicate ±1 standard deviation.

Soybean exhibited distinct characteristics in the reflectance and VI time-series compared to other crops (Fig. 7). For the Blue, Green, Red, and Red Edge 1 bands (Fig. 7a–d), both soybean and maize had lower reflectance than other crops. However, soybean showed higher reflectance than maize in the Red Edge 2, Red Edge 3, and NIR bands (Fig. 7e-g), as well as in the SWIR bands during the late growing season (Fig. 7h-i). Among VIs, soybean was particularly distinguishable based on GWCCI2 and NDSVI (Fig. 7l, n), which exhibited the highest values during the peak growing season as well as rapid increase and decrease. As the most commonly cultivated crop in soybean-growing regions, maize exhibited consistently higher REP values than other crops throughout the growing season (Fig. 7p). These characteristics aligned well with the optimised features (Table 3), although substantial intra-class variations (i.e., shaded areas in Fig. 7) were also observed.

### Classification accuracies for different years

Accuracy assessment based on samples from all four years showed that the classification model achieved good performance, with an OA of 0.89, an F1-score of 0.66 for the soybean class, and an F1-score of 0.94 for the non-soybean class (Table 5). Among the four years, soybean accuracies in 2020 and 2021 (F1-scores of 0.71 and 0.77, respectively) were higher than in 2018 and 2019 (0.62 and 0.60, respectively), while the non-soybean class consistently maintained high accuracy (F1-scores above 0.90). Comparing the precision and recall metrics, the soybean class exhibited higher precision than recall across all four years, with larger differences in the lower-accuracy years (2018 and 2019) than in the higher-accuracy years (2020 and 2021). In addition, LOYO and LORO showed comparable accuracies to the train-test-split validation, with slight decreases in the soybean F1-score of 0.02 and 0.01, respectively (Table 5).

**Table 5:** Model classification accuracy for different years and validation results for leave-one-year-out (LOYO) and leave-one-region-out (LORO) experiments.

Year	Class	Precision	Recall	F1-score	OA
2018	Soy	0.72 [0.63, 0.82]	0.55 [0.45, 0.65]	0.62 [0.54, 0.70]	0.89 [0.87, 0.92]
	Non-soy	0.92 [0.90, 0.94]	0.96 [0.94, 0.98]	0.94 [0.93, 0.95]	
2019	Soy	0.68 [0.61, 0.74]	0.54 [0.47, 0.61]	0.60 [0.54, 0.66]	0.86 [0.84, 0.88]
	Non-soy	0.89 [0.88, 0.91]	0.94 [0.92, 0.95]	0.92 [0.90, 0.93]	
2020	Soy	0.74 [0.66, 0.82]	0.68 [0.59, 0.77]	0.71 [0.63, 0.78]	0.91 [0.89, 0.93]
	Non-soy	0.94 [0.92, 0.96]	0.95 [0.93, 0.97]	0.95 [0.93, 0.96]	
2021	Soy	0.80 [0.72, 0.87]	0.75 [0.65, 0.83]	0.77 [0.70, 0.83]	0.93 [0.91, 0.95]
	Non-soy	0.95 [0.94, 0.97]	0.96 [0.95, 0.98]	0.96 [0.95, 0.97]	
All	Soy	0.71 [0.67, 0.75]	0.61 [0.57, 0.66]	0.66 [0.62, 0.69]	0.89 [0.88, 0.90]
	Non-soy	0.92 [0.91, 0.93]	0.95 [0.94, 0.96]	0.94 [0.93, 0.94]	
All (LOYO)	Soy	0.68 [0.63, 0.73]	0.60 [0.51, 0.69]	0.64 [0.57, 0.71]	0.89 [0.86, 0.92]
	Non-soy	0.93 [0.91, 0.95]	0.94 [0.93, 0.95]	0.93 [0.91, 0.95]	
All (LORO)	Soy	0.64 [0.61, 0.67]	0.66 [0.58, 0.74]	0.65 [0.61, 0.69]	0.90 [0.89, 0.91]
	Non-soy	0.94 [0.93, 0.95]	0.94 [0.93, 0.95]	0.94 [0.93, 0.95]	

*Values in brackets for individual years represent 95% confidence intervals, while those for LOYO and LORO represent the standard deviations of the accuracy metrics.

### Classification accuracies for different field types

Based on water stress levels, crop samples from all four years were categorised into four types, and the classification accuracies were assessed for each field type (Table 6). Accuracies for Type I (no water stress) and Type II (mildly water-stressed) were higher than the overall average, whereas those for Type III (moderately water-stressed) and Type IV (severely water-stressed) were lower. Specifically, with increasing water stress, classification accuracies for both soybean and non-soybean classes gradually decreased. Comparing Type I and Type IV, the OA decreased from 0.92 to 0.75, and the F1-scores of soybean and non-soybean decreased from 0.79 to 0.51 and from 0.95 to 0.83, respectively. These variations in soybean accuracy across field types were mainly due to the changes in recall, while precision remained relatively stable.

**Table 6:** Model classification accuracy for different field types.

Field	Class	Precision	Recall	F1-score	OA
Type I	Soy	0.75 [0.69, 0.81]	0.84 [0.78, 0.89]	0.79 [0.74, 0.84]	0.92 [0.90, 0.94]
	Non-soy	0.96 [0.95, 0.97]	0.94 [0.91, 0.95]	0.95 [0.94, 0.96]	
Type II	Soy	0.79 [0.71, 0.87]	0.59 [0.50, 0.68]	0.68 [0.60, 0.75]	0.86 [0.84, 0.89]
	Non-soy	0.88 [0.86, 0.90]	0.95 [0.93, 0.97]	0.91 [0.90, 0.93]	
Type III	Soy	0.71 [0.63, 0.80]	0.54 [0.45, 0.63]	0.61 [0.53, 0.69]	0.82 [0.78, 0.85]
	Non-soy	0.84 [0.82, 0.87]	0.92 [0.89, 0.95]	0.88 [0.86, 0.90]	
Type IV	Soy	0.72 [0.61, 0.83]	0.40 [0.30, 0.50]	0.51 [0.42, 0.60]	0.75 [0.71, 0.79]
	Non-soy	0.76 [0.73, 0.79]	0.92 [0.89, 0.96]	0.83 [0.81, 0.86]	

*The values in brackets [] represent the 95% confidence intervals for the accuracy metrics.

### Soybean spatial distribution

**Fig. 8:**
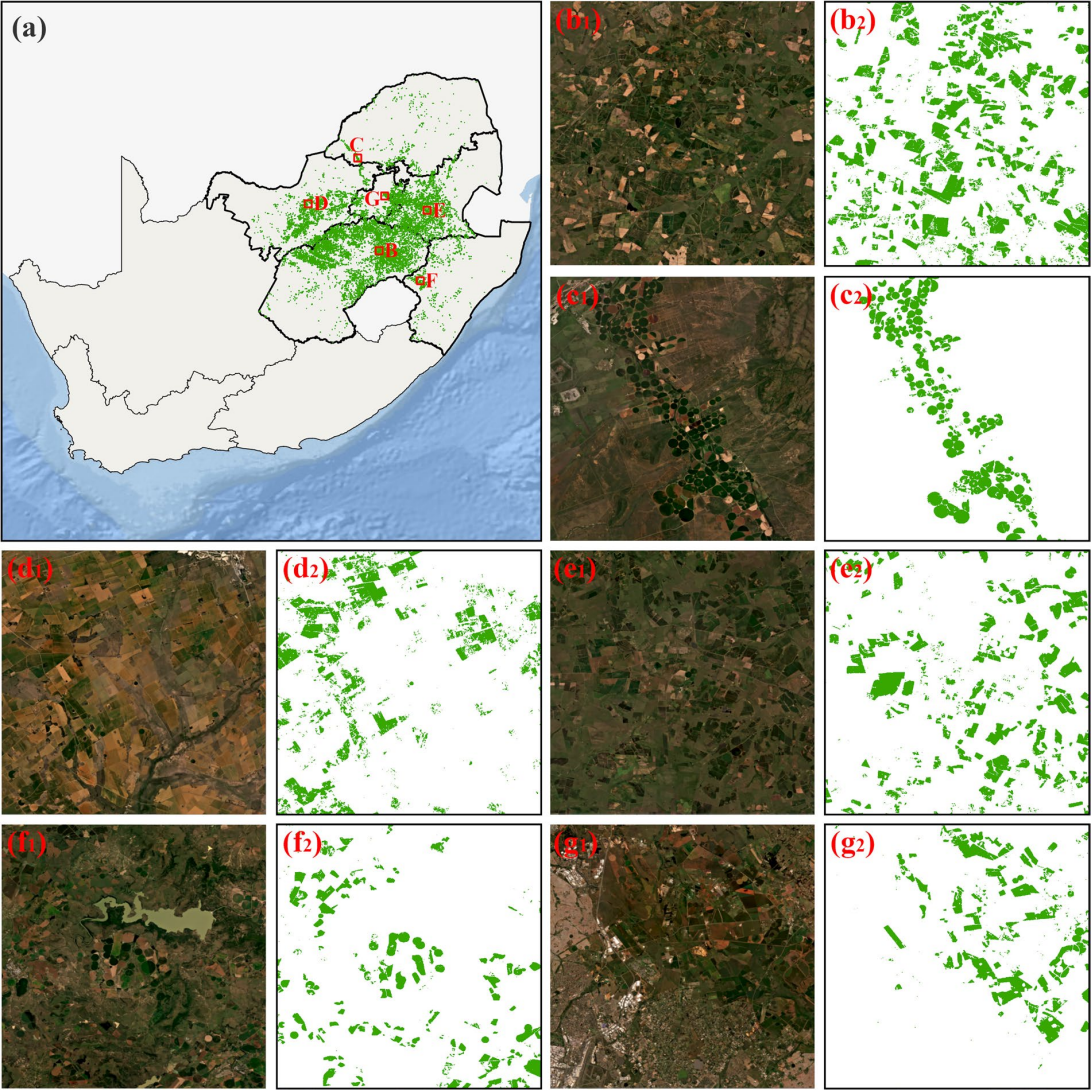
Spatial distribution of soybean cultivation in 2024 (**a**), compared against Planet monthly basemaps during the soybean peak growing season (January to March) at the zoom-in views (20 km × 20 km) of typical landscapes in Free State (**b**), Limpopo (**c**), North West (**d**), Mpumalanga (**e**), KwaZulu-Natal (**f**), and Gauteng (**g**).

The soybean map for 2024 (Fig. 8a) showed that cultivation was concentrated in northern Free State, south-western Mpumalanga, south-eastern North West, and adjoining areas of neighbouring provinces, forming a core cultivation region. Visual comparison with Planet monthly basemaps from the soybean peak growing season (January to March) at zoom-in views indicated that the classified soybean were mostly located within croplands (Fig. 8b–g), with clear separation from non-cropland covers such as natural vegetation (Fig. 8b, c, e), water bodies (Fig. 8f), and built-up areas (Fig. 8g).

### Comparison with the reported area at the provincial level

**Fig. 9:**
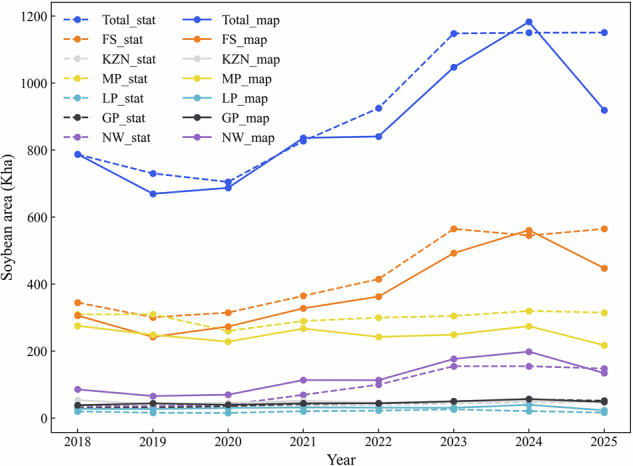
Comparison of temporal trends between reported and mapped soybean areas. FS: Free State, KZN: KwaZulu-Natal, MP: Mpumalanga, LP: Limpopo, GP: Gauteng, and NW: North West.

The comparison between reported and mapped areas showed that SoySA10 effectively captured soybean cultivation dynamics from 2018 to 2025 at both national and provincial levels (Fig. 9). Statistics indicated that South Africa experienced rapid soybean expansion from 2020 to 2023, with cultivated area increasing from an average of 741 Kha (2018–2020) to 1,150 Kha (2023–2025). Correspondingly, SoySA10 mapped an increase from 715 Kha to 1,050 Kha, closely matching the national reported areas. Among the six provinces, FS and NW were the leading contributors to this expansion, while cultivated areas in other provinces remained relatively stable.

**Fig. 10:**
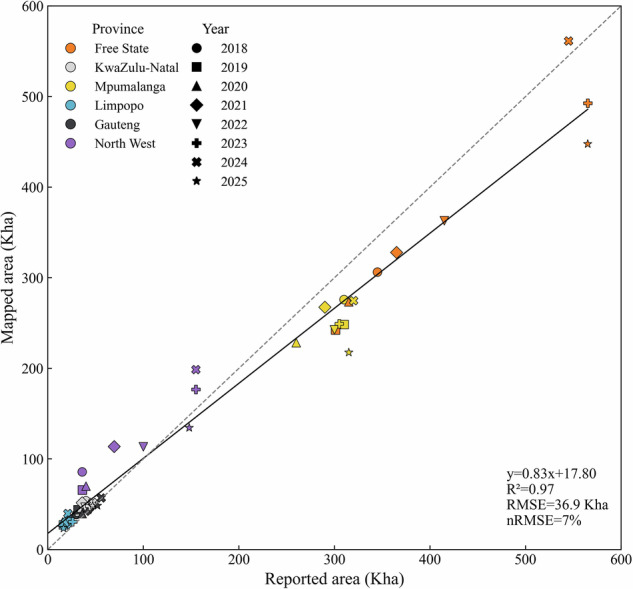
Scatter plot of reported and mapped areas at the provincial level across different years. The variant of nRMSE here is nRMSE_s_.

Scatter plots further confirmed the high overall consistency between the reported and mapped areas at the provincial level (Fig. 10). Compared with the reported areas, the mapped area achieved an R² of 0.97 and an (n)RMSE of 36.9 Kha (7%) when combining all years and provinces. For individual years (Table 7), the areas in 2020, 2021, and 2024 showed higher consistency, with RMSE values below 30 Kha, whereas the RMSE values for 2019, 2023, and 2025 exceeded 35 Kha. For individual provinces (Table 7), major producing provinces (i.e., Free State and Mpumalanga) showed higher consistency, with nRMSE below 20%, while North West and Limpopo showed relatively larger discrepancies, with nRMSE values of 36% and 57%, respectively.

**Table 7:** (n)RMSE of reported and mapped areas^*^.

Year	(n)RMSE/Kha (%)	Province	(n)RMSE/Kha (%)
2018	30.2 (9%)	Free State	61.3 (14%)
2019	37.6 (13%)	KwaZulu-Natal	9.5 (24%)
2020	25.8 (9%)	Mpumalanga	55.3 (18%)
2021	26.5 (8%)	Limpopo	11.3 (57%)
2022	32.5 (8%)	Gauteng	5.8 (14%)
2023	38.4 (7%)	North West	33.4 (36%)
2024	27.7 (5%)		
2025	62.6 (11%)		

Left: Annual results (8 years) across all provinces. Right: Provincial results (6 provinces) across all years.

^*^The variants of nRMSE for each year and each province are nRMSE_t_ and nRMSE_s_, respectively.

### Comparison with the reported area at the district level

**Fig. 11:**
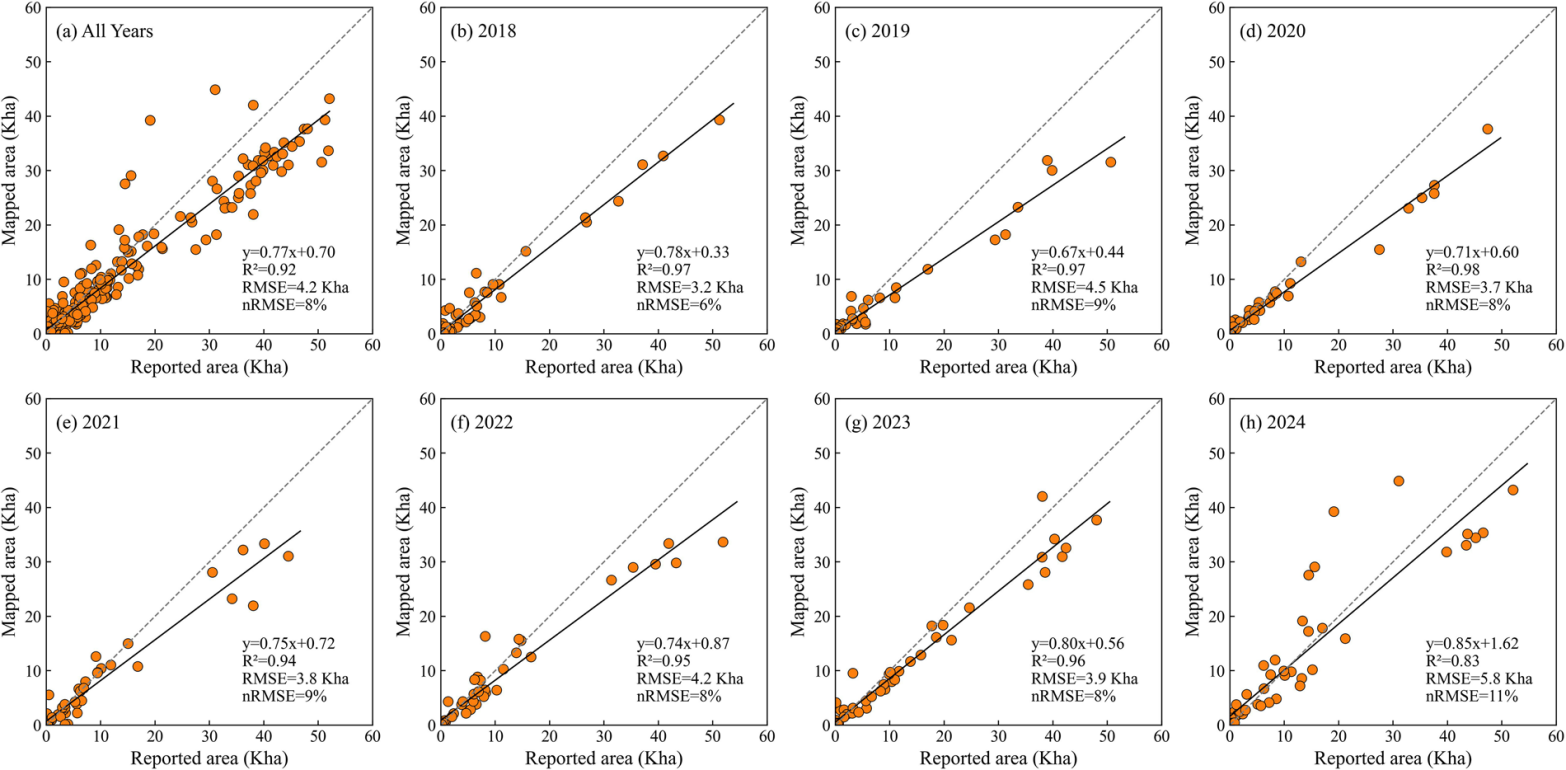
Scatter plot of reported and mapped areas at the district level (52 districts) across different years for Free State Province. The variant of nRMSE here is nRMSE_s_.

**Fig. 12:**
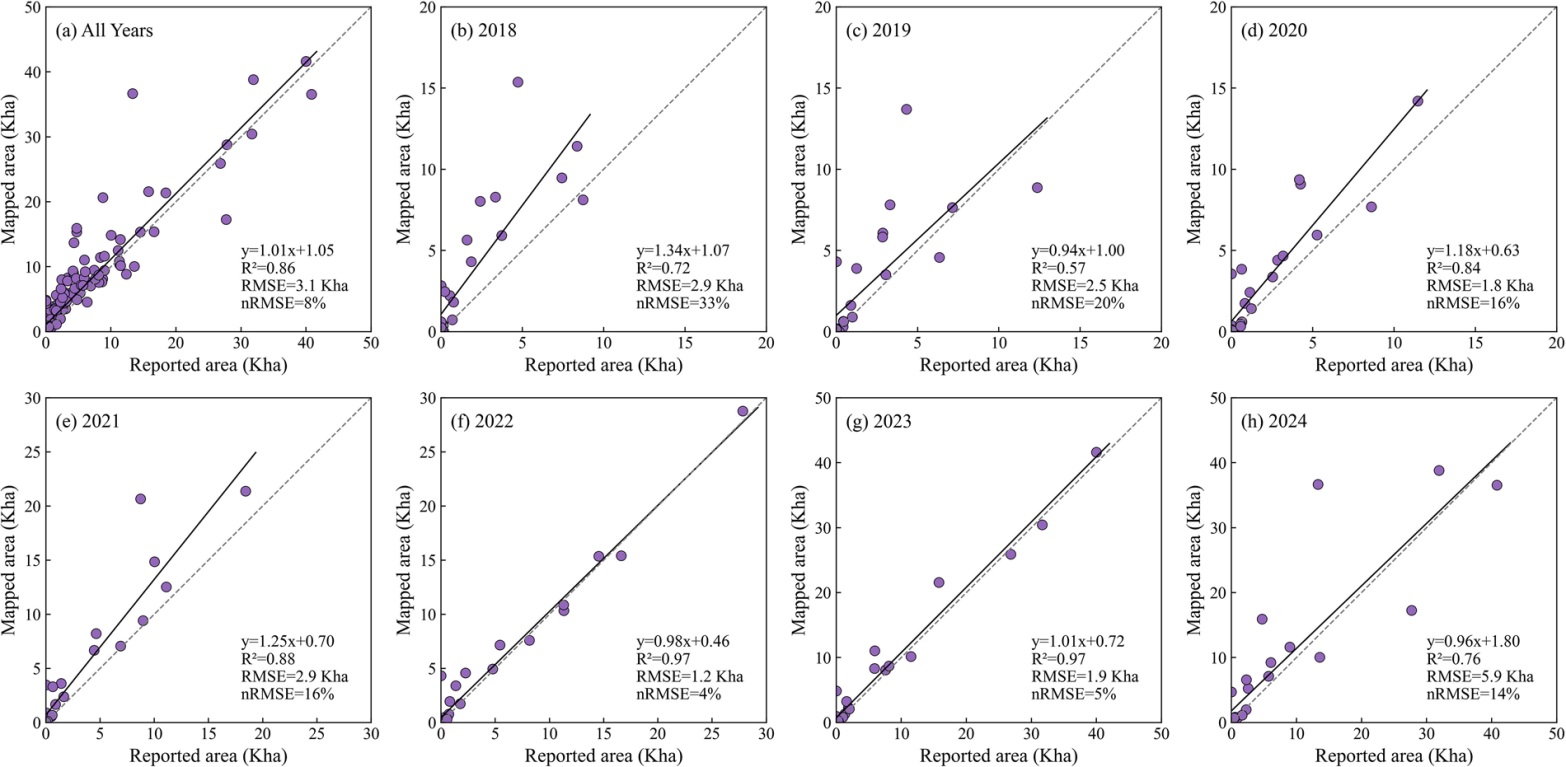
Scatter plot of reported and mapped areas at the district level (27 districts) across different years for North West Province. The variant of nRMSE here is nRMSE_s_.

Compared with the provincial level, the consistency between the reported and mapped areas decreased slightly at a finer spatial scale (i.e., district level) for FS and NW provinces (Figs. 11, 12, respectively) but remained high (Table 8). When combining all years, the R^2^ values for FS and NW provinces were 0.92 and 0.86, respectively, with (n)RMSE values of 4.2 Kha (8%) and 3.1 Kha (8%). For FS province, the consistency in 2019, 2022, and 2024 was lower than in other years, with RMSE exceeding 4 Kha (~10%). Similarly, 2018, 2019, and 2024 were lower-accuracy years for NW province, with R^2^ values of 0.72, 0.57, and 0.76, and nRMSE values of 33%, 20%, and 14%, respectively.

**Table 8:** Summary of R^2^ and nRMSE_s_ for reported and mapped areas at provincial and district levels*.

Accuracy metric	Provincial level	District level (FS)	District level (NW)
R^2^	0.97	0.92 (0.83–0.98)	0.86 (0.57–0.97)
nRMSE_s_	7% (5%–13%)	8% (6%–11%)	8% (4%–33%)

*FS: Free State; NW: North West. Values outside parentheses represent the aggregate across all years; values within parentheses indicate the range across individual years.

### Comparison with the SPAM 2020 map

**Fig. 13:**
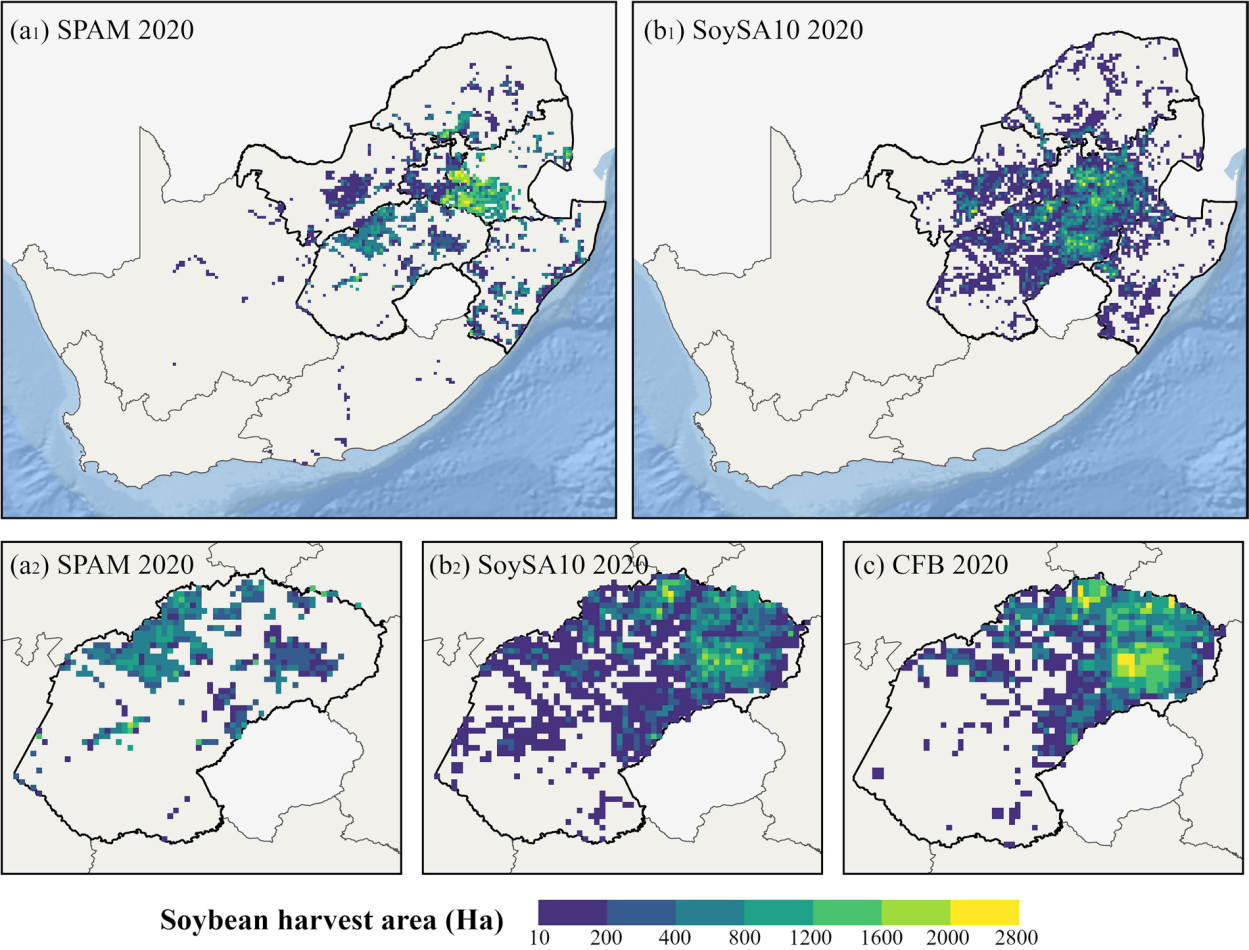
Comparison of overall soybean spatial distribution across South Africa between SPAM 2020 (**a**_**1**_) and SoySA10 2020 (**b**_**1**_), and within Free State among SPAM 2020 (**a**_**2**_), SoySA10 2020 (**b**_**2**_), and the crop field boundary (CFB) dataset (**c**). The soybean harvest areas from SoySA10 and the CFB dataset were aggregated to the 10 km scale for consistency with SPAM.

SPAM 2020 and SoySA10 2020 exhibited similar overall spatial distribution patterns of soybean cultivation across South Africa (Fig. 13a_1_, b_1_). Specifically, both datasets identified the northern Free State, the south-western Mpumalanga, and the south-eastern North West as the major soy-cultivating regions, and the overall distributions in the remaining three provinces also showed good agreement. Compared with SPAM 2020, SoySA10 2020 indicated more areas with soybean cultivation, particularly in north-eastern Free State. To further evaluate the results, the soybean harvest area from the CFB dataset in 2020 for Free State was aggregated at the same 10 km scale and used as a more accurate reference. The comparison suggested that SoySA10 more accurately captured the overall soybean distribution in Free State than SPAM in 2020 (Fig. 13a_2_, b_2_, c).

### Comparison with the CFB dataset in free state

**Fig. 14:**
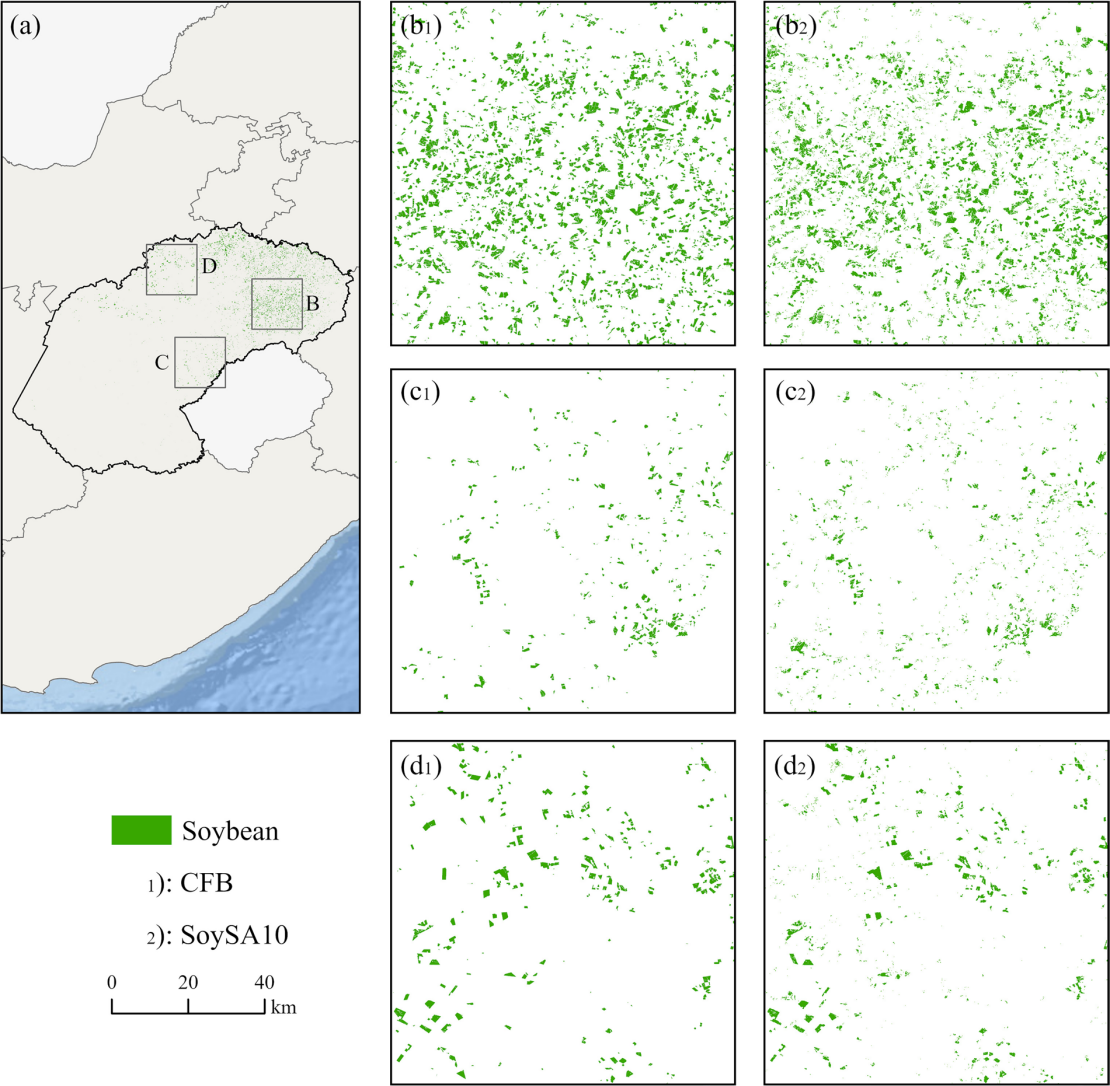
Detailed spatial comparison between the crop field boundary (CFB) dataset and SoySA10. Panel (**a**) shows the overall soybean distribution in the CFB dataset, and panels (**b**–**d**) present zoom-in views at 80 km × 80 km.

Detailed soybean distributions from SoySA10 were compared with the CFB dataset for Free State in 2020 (Fig. 14), which provides field-level geometry data and served as the reference. The comparison showed good spatial consistency between the two datasets in both concentrated cultivation regions (Fig. 14b) and sparse cultivation regions (Fig. 14c, d), demonstrating the strong reliability of SoySA10 in providing spatially detailed information on soybean cultivation.

### Soy-cultivation change map

**Fig. 15:**
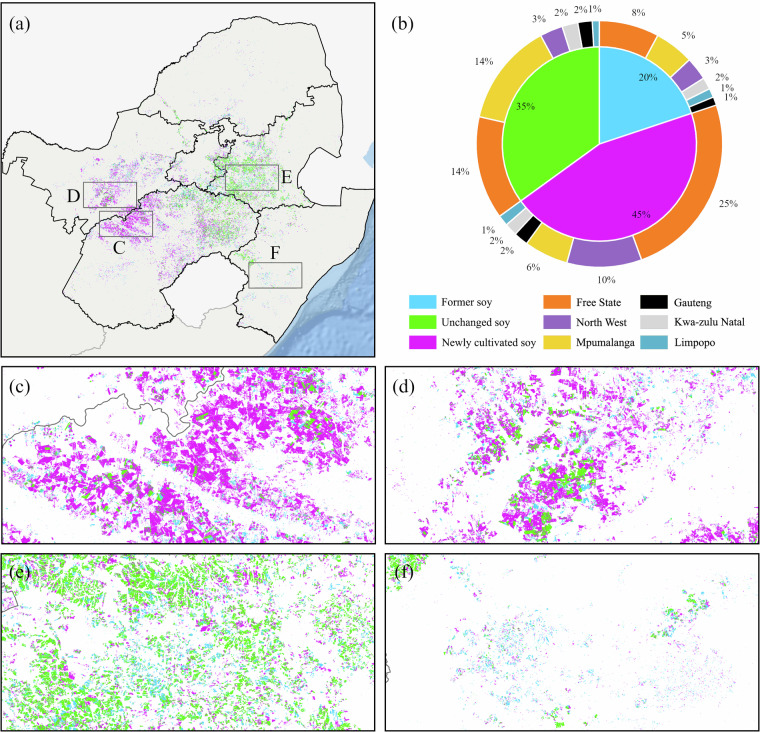
Soy-cultivation change map between 2018–2020 and 2023–2025 (**a**); proportions of different change types and their distribution across provinces (**b**); zoom-in views of frontiers of soybean increase in Free State (**c**) and North West (**d**), stable soybean cultivation regions in Mpumalanga (**e**), and regions with soybean decrease in KwaZulu-Natal (**f**).

The produced soy-cultivation change map provides spatially detailed information on soybean dynamics across South Africa and exhibited consistent dynamics with statistics (Fig. 15). Between the periods 2018–2020 and 2023–2025, 45% of soybean fields were newly cultivated, primarily in Free State (25%) and North West (10%); 35% of soybean fields remained unchanged, mainly in Free State (14%) and Mpumalanga (14%); and 20% of soybean fields were formerly cultivated, mostly in Free State (8%) and Mpumalanga (5%). Spatially, soybean cultivation expanded mainly in the north-western areas of Free State (Fig. 15c) and the south-western areas of North West (Fig. 15d). In contrast, Mpumalanga was predominantly stable, with the majority of soybean fields remaining unchanged, interspersed with both newly cultivated and former fields (Fig. 15e). Regions dominated by former soybean fields were identified, such as in KwaZulu-Natal, where many small fields were no longer cultivated with soybean (Fig. 15f).

### Soybean field type map

**Fig. 16:**
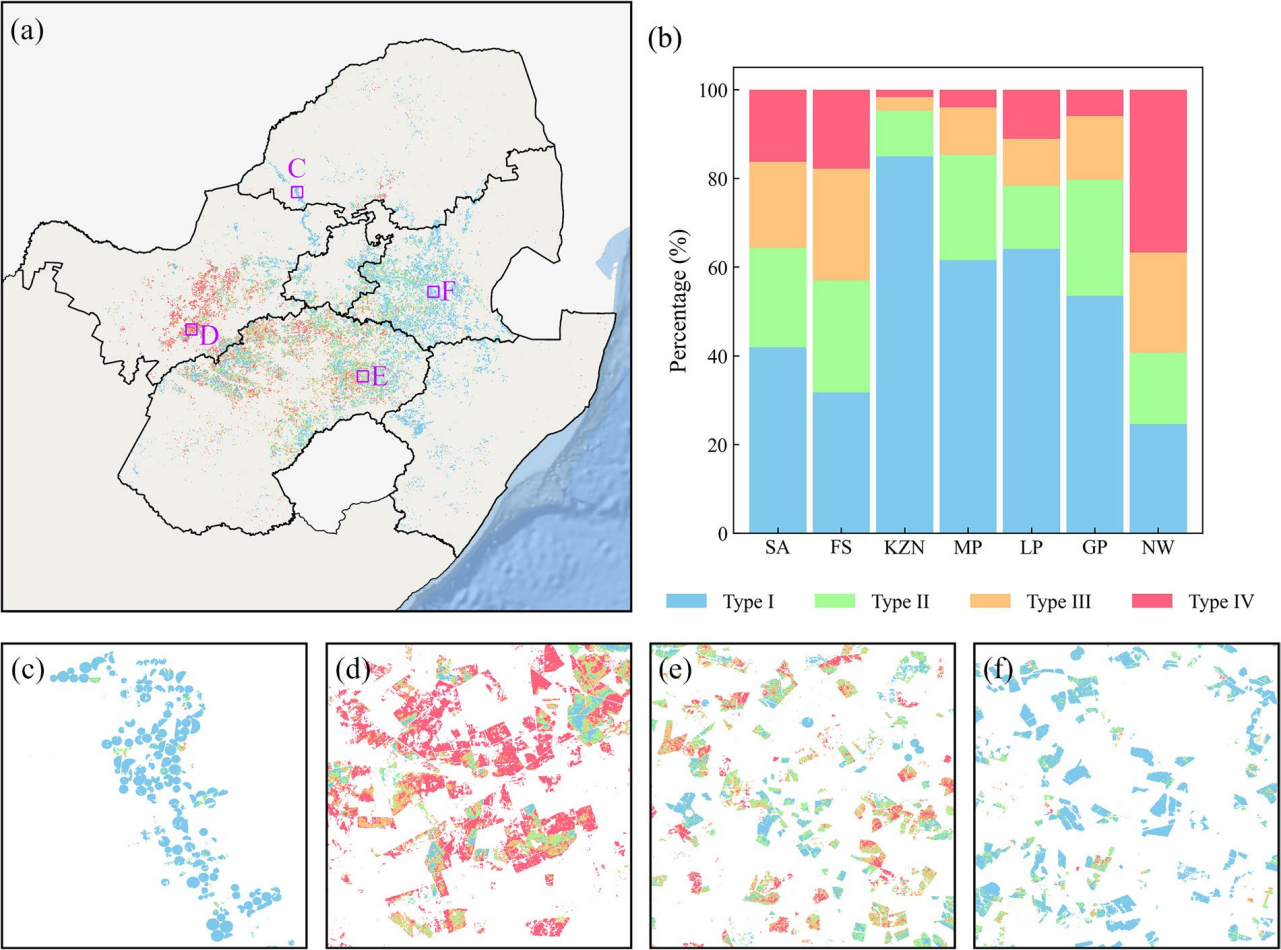
Soybean field type map from SoySA10 in 2024 (**a**); proportions of different field types in South Africa and its provinces (**b**); zoom-in views of field types for pivots in Limpopo (**c**), expansion frontiers in North West (**d**), and cultivated areas in Free State (**e**) and Mpumalanga (**f**). SA: South Africa, FS: Free State, KZN: KwaZulu-Natal, MP: Mpumalanga, LP: Limpopo, GP: Gauteng, and NW: North West.

The soybean field type map for 2024 revealed significant spatial variation in field type distributions across different provinces, and accuracy variations were consistent with the proportions of water-stressed field types (Fig. 16). For example, types III and IV together accounted for 60% of soybean fields in North West and 43% in Free State, which was in accordance with the lower accuracy observed in 2024, especially for the North West province. In contrast, type I represented more than 80% of soybean fields in KwaZulu-Natal. Types I and II together comprised about 80% of soybean fields in Mpumalanga, Limpopo, and Gauteng. Water-stressed soybean fields (i.e., types III and IV) were mainly located in south-western North West and north-western Free State, largely overlapping with areas previously identified as soybean expansion frontiers (Fig. 15). In contrast, pivot soybean fields (Fig. 16c) and unchanged soybean fields (Fig. 16f) were more likely to experience no or mild water stress (i.e., types I and II).

### Limitations and prospects

This study designed an effective framework for large-scale soybean mapping in South Africa, an emerging and major soybean producing country in Africa. However, several limitations of SoySA10 need to be acknowledged, and future work could be considered to further improve the dataset. First, the only satellite data source used for mapping was Sentinel-2. Integrating additional sources, such as Sentinel-1 and Landsat-8/9, could provide denser observations and complementary information, including radar-derived features that are sensitive to water content and crop structure^45,46^. Moreover, recently released artificial intelligence foundation datasets, such as Google AlphaEarth Foundations^47^ and TESSERA^48^, could be explored, as they have shown strong generalisation ability and good performance in RS-related tasks. Second, although soybean fields were categorised into different field types to provide supplementary information on mapping accuracy, classification performance could be further improved for the more challenging types (i.e., Types III and IV). Accuracy for these types could be enhanced by identifying type-specific classification features (i.e., distinct soybean features under water stress) or by designing features that are invariant to crop performance or water stress^49,50^. Third, since crop samples were collected only from the Free State, the reported model accuracies are not fully representative of the map accuracies across South Africa, although other independent data sources were used for validation. Given the challenges of conducting extensive ground surveys nationwide, users are encouraged to independently validate our products in their regions of interest and provide feedback for future improvements. In addition, the mapped areas were directly derived from SoySA10 for consistency comparisons with reported areas, which could be biased. For users aiming to derive precise area estimates from our maps, we recommend following the guidelines of Olofsson *et al*.^51^ for unbiased area estimation. Lastly, as the second-largest soybean producer in Africa with a rapidly expanding cultivated area, South Africa was selected as a representative country for soybean mapping. Future work could be extended to other major soy-producing countries in Africa if sufficient crop type samples are available. Crop type classification in smallholder systems is limited by a lack of adequate and precise crop labels that are uniformly distributed across different environmental gradients. Recent initiatives have demonstrated the strength of citizen science platforms for rapid and cost-effective collection of crop labels by mounting the GoPro cameras on the helmets of motorbike riders, which is a key means of transport in the rural villages^52^. Such initiatives are expected to address the scarcity of crop label data in Africa. Nonetheless, additional improvements or adaptations of our classification method may be required when applied to more challenging contexts, such as smaller field sizes, higher diversity of cultivated crops, and sub-optimal crop management.

## Data Availability

The produced soybean dataset (SoySA10) and the reference crop label dataset for South Africa are publicly available on Zenodo (10.5281/zenodo.17569053).

## Code availability

The dataset (SoySA10) was generated using Google Earth Engine (GEE). Fully reproducible code is available via the GEE repository at https://code.earthengine.google.com/?accept_repo=users/huangxgeo/SoySA10 (accessible to users with a GEE account) and on GitHub at https://github.com/huangxgeo/SoySA10_GEE, which does not require a GEE account.
